# Analysis of the spleen proteome of chickens infected with reticuloendotheliosis virus

**DOI:** 10.1007/s00705-016-3180-5

**Published:** 2017-01-17

**Authors:** Mei Xue, Yan Zhao, Shunlei Hu, Xingming Shi, Hongyu Cui, Yunfeng Wang

**Affiliations:** 1 State Key Laboratory of Veterinary Biotechnology, Harbin Veterinary Research Institute, The Chinese Academy of Agricultural Sciences, Harbin, 150001 People’s Republic of China; 2 National Engineering Research Center of Veterinary Biologics, Harbin, People’s Republic of China

**Keywords:** Gene Ontology, Ferritin, Protein Spot, Severe Acute Respiratory Syndrome, Infectious Bursal Disease Virus

## Abstract

Infection with reticuloendotheliosis virus (REV), a gammaretrovirus in the family *Retroviridae*, can result in immunosuppression and subsequent increased susceptibility to secondary infections. In the present study, we identified differentially expressed proteins in the spleens of chickens infected with the REV-A HLJ07I strain, using two-dimensional gel electrophoresis on samples from time points coinciding with different phases of the REV life cycle. Differentially expressed proteins were identified using one-dimensional liquid chromatography electrospray ionization tandem mass spectrometry (1D LC ESI MS/MS). Comparative analysis of multiple gels revealed that the majority of changes occurred at early stages of infection. In total, 60 protein spots representing 28 host proteins were detected as either quantitatively (false discovery rate [FDR] ≤0.05 and fold change ≥2) or qualitatively differentially expressed at least once during different sampling points. The differentially expressed proteins identified in this study included antioxidants, molecular chaperones, cellular metabolism, formation of the cytoskeleton, signal transduction, cell proliferation and cellar aging. The present findings provide a basis for further studies to elucidate the role of these proteins in REV-host interactions. This could lead to a better understanding of REV infection mechanisms that cause immune suppression.

## Introduction

Reticuloendotheliosis viruses (REVs) are a group of viruses in the family *Retroviridae*, specifically, gammaretroviruses in the same genus as mammalian C-type retroviruses [12]. The REV group includes defective REV-T [32, 33], non-defective REV-A [11, 75], chick syncytial virus [14], duck infectious anemia virus [41], and spleen necrosis virus (SNV) [66]. REVs cause immunosuppression, runting disease, and lymphoma in a variety of avian hosts, including chickens, turkeys, ducks, geese, pheasants, peafowl, and some other bird species [7]. The immunosuppression caused by REV infection increases the susceptibility to concurrent or secondary bacterial or viral infections and results in poor immune responses to other vaccines [38, 80]. REV can co-infect with other viruses [17] and cause contamination of a variety of poultry biologics [31]. In addition, REV integrates easily into the host genome, and it has been associated with a number of hematopoietic cell tumors [37, 76]. Therefore, REV poses a serious threat to the poultry industry.

Knowledge of the interactions between virus and host is critical for understanding the pathogenesis of viral infection. The expression of host cellular proteins changes greatly during retroviral infection, from the entry of the virus to incorporation of viral DNA into the host genome, and finally to the release of virions. By applying comparative proteomics techniques, changes in the expression profile of cellular proteins during viral infections can be monitored, which may help to further elucidate viral pathogenesis [63]. Proteomics studies using a human immunodeficiency virus (HIV)-infected monocyte/macrophage lineage were the first to be applied in a retrovirus infection [39]. However, the utilization of two-dimensional gel electrophoresis (2-DE) to investigate the proteomic changes that occur in REV-A-infected chickens has not been reported.

The present study was designed to examine the spleen proteome of specific-pathogen-free (SPF) chickens in the course of infection with an REV-A strain. To address this objective, several time points were selected for harvesting tissues that corresponded to important phases of REV pathogenesis. A total of 28 differentially expressed proteins were identified. Analyses and functional studies of these proteins will provide novel information for better understanding of the pathogenesis of REV and the mechanisms of virus-host interactions.

## Materials and methods

### Experimental animals and the virus strain used for infection

All of the chickens used in this experiment were one-day-old specific-pathogen-free (SPF) White Leghorn chickens obtained from Harbin Veterinary Research Institute, Chinese Academy of Agricultural Sciences. Chickens were kept in isolators at Harbin Veterinary Research Institute throughout the experiment.

Chickens were infected with the HLJ07I strain of REV-A (GenBank accession no. GQ375848), which was isolated in Heilongjiang Province of China in 2007. REV was propagated in chicken embryo fibroblast (CEF) as described previously [77].

### Experimental design

Fifty-four one-day-old SPF chickens were randomly divided into two groups and were housed in isolators. One group of chickens (n = 27) was inoculated intra-abdominally with 10^4.6^ tissue culture infective doses 50% (TCID_50_) of the REV-A HLJ07I strain on day 3 after hatching. The rest (n = 27) were kept as uninfected controls. Infected and uninfected control chickens were kept in separate isolators with similar environmental conditions. All chickens had free access to feed and water during the rearing period.

Nine chickens were selected randomly from each group and killed humanely at 7, 14, and 21 days post-inoculation. Spleens were separated rapidly and washed with ice-cold phosphate-buffered saline (PBS) buffer, snap-frozen in liquid nitrogen, and kept subsequently at -80 °C until further processing. Animal experiments were performed in accordance with animal ethics guidelines and approved protocols. All animal studies were approved by the Animal Ethics Committee of Harbin Veterinary Research Institute of the Chinese Academy of Agricultural Sciences (SYXK (Hei) 2011022).

### Quantitative analysis of REV in spleen by real-time RT-PCR

Tissue samples were ground and homogenized, 100 mg of tissue homogenate was suspended in 500 μl of PBS containing 100 μg of penicillin and 100 μg of streptomycin per ml, and the suspension was frozen and thawed three times and centrifuged at 13,000×*g* at 4 °C for 5 minutes. RNA was extracted from 200 μl of tissue supernatant using TRIzol Reagent (Invitrogen) following the manufacturer’s instructions. The amount of total RNA in each sample was determined using a NanoVue Spectrophotometer. cDNA was synthesized, using AMV reverse transcriptase (TaKaRa) and 0.5 μg of random primers (TaKaRa), at 42 °C for 60 min. The cDNA was stored at −20 °C until it was used in the real-time PCR. The absolute REV genome load in the REV-infected spleens of chickens was quantified using primers specific for REV gag gene. The primers used are as follows: forward primer, AGACTCGCATTGTCGATGTCTTG; reverse primer, CAAATCTTTGCCAATCAA TATCAG. Linear regression analysis of the standard curve was used to estimate the number of viral genomic RNA copies per 100 ng of spleen RNA. The standard RNA curve was linear in the range between 10^2^ molecules at the lower limit and 10^9^ molecules at the upper limit. A real-time PCR assay was performed in a total volume of 20 μl containing 10 μl of SYBR^®^ Premix Ex Taq^TM^ (2×; Takara, Shiga, Japan), 100 ng of cDNA, 10 pmol of forward primer, and 10 pmol of reverse primer using a LightCycler^®^ 480 Real-Time PCR System (Roche Diagnostics). The PCR protocol consisted of an initial denaturation step at 95 °C for 120 s and 40 cycles of denaturation (95 °C for 15 s), annealing (61 °C for 30 s), and extension (72 °C for 15 s). For each step, the temperature transition rate was 20 °C/s. Experiments on each sample were performed in triplicate with the above primers. The data were analyzed using LightCycler^®^ 480 Software Version 1.5.

### Sample preparation for proteomic analysis

The frozen tissues were rinsed in ice-cold PBS buffer and then placed in liquid nitrogen and ground thoroughly to a very fine powder. Tissue powder (100 mg) was dissolved in 500 μl of lysing solution containing 7 M urea, 2 M thiourea, 4% CHAPS, 40 mM DTT, 2% IPG buffer, pH 3-10 or pH 4-7, 1% Nuclease Mix and 1% Protease Inhibitor Mix (GE Healthcare, Amersham, UK), incubated for 2 h at room temperature with vortexing once every 15 min, and centrifuged at 15000×*g* for 1 h at 4 °C. The supernatant was collected and purified using a Plus One 2-D Clean-up kit (GE Healthcare, Amersham, UK). The concentration of each protein sample was determined using a Plus One 2-D Quant Kit (GE Healthcare). Protein samples were aliquoted and stored at -80 °C for 2-DE analysis.

### Two-dimensional polyacrylamide gel electrophoresis (2D-PAGE)

Three independent sample pools of each kind of tissue per group were used for 2-DE analysis. Each analytical 2D-PAGE gel was prepared with 400 μg of protein mixed with rehydration buffer (8 M urea, 2% CHAPS, 90 mM DTT, 5 μl of the appropriate IPG buffer per ml, 12 μl of DeStreak Reagent (GE Healthcare) per ml and 0.005% bromophenol blue) to a total volume of 250 μl. The first-dimension separation was performed in 24-cm, pH 4-7 non-linear Immobiline DryStrips (GE Healthcare) using an Ettan IPGphor isoelectric focusing unit (GE Healthcare). After rehydration at 30 V for 12 h, isoelectric focusing was performed at 500 V for 1 h, 1000 V for 1 h and 8000 V until a total of 57,000 volt hours was reached. Each focused strip was incubated at room temperature, initially in 10 ml of equilibration buffer (50 mM Tris-Cl [pH 8.8], 6 M urea, 30% [v/v] glycerol, 2% [w/v] SDS and 0.005% bromophenol blue) containing 1% (w/v) DTT for 15 min and subsequently in a similar volume of equilibration buffer containing 2.5% (w/v) iodoacetamide for a similar time. For the second-dimension separation, each IPG strip was placed on a 12.5% SDS-polyacrylamide gel, and six such gels were simultaneously run each time subjecting each gel to 25 mA of current at 25 °C in the SE600 Ruby system (GE Healthcare) until the bromophenol blue dye front reach the opposite edge of the gel. Each gel was subsequently fixed for 1 h in a solution containing 10% (v/v) methanol and 7% (v/v) acetic acid. Then, the gels were stained with PlusOne Coomassie Blue R-350 (GE Healthcare) and scanned using an Image Scanner III (GE Healthcare). Quantitative analysis was performed using Image Master 2D Platinum software v6.0 (GE Healthcare). For image analysis, three independent gels from the REV-infected group were compared with those from the corresponding control group at 7, 14, and 21 days postinfection (dpi). The normalized volume values (vol %) of matched protein spots were subjected to Student’s *t*-test using the SPSS statistical software package version 16.0. The criterion used to define differential expression of spots was that the ratio of the vol % in the REV-infected group vs. the control group was more than 1.5 (*p* < 0.05) or less than 0.67 (*p* < 0.05). Protein spots corresponding to differentially expressed proteins were excised manually from the gels and subjected to mass spectrometry analysis.

### MALDI-TOF/TOF MS and database search

Gel samples were placed in a tube and washed once with 500 μl of ddH_2_O and once with 250 μl of ddH_2_O, each for 15 min. For trypsin digestion, the gel samples were washed twice with 50 mM NH_4_HCO_3_ and covered with 50 mM NH_4_HCO_3_ containing10 mg of porcine trypsin solution (Promega, Madison, WI, USA) per ml. After incubation overnight at 37 °C, the supernatant was transferred to a second tube, and 40 μl of 50 mM NH_4_HCO_3_ was added. Gel samples were washed with 40 μl of 50 mM NH_4_HCO_3_, and the supernatants were collected and combined. The gel was washed with 70% v/v acetonitrile (ACN) and dried in a SpeedVac Vacuum Concentrator (Bachhofer). The peptide mixtures were desalted using ZipTip C-18 RP tips (Millipore, Billerica, MA, USA), which were wetted with 100% ACN and equilibrated with 0.1% trifluoroacetic acid (TFA). Peptide samples, which were redissolved in 10 ml of 0.5% TFA, were eluted with 50% ACN/0.1% TFA and dried in a SpeedVac Vacuum Concentrator.

The purified peptides were spotted on a MALDI plate and covered with 0.7 μl of 2 mg/ml 3, 5-dimethoxy-4-hydroxycinnamic acid matrix (Sigma, St. Louis, MO, USA) with 10 mM NH_4_H_2_PO_4_ in 60% ACN. All samples were analyzed by MALDI-TOF/TOF MS using a 4700 Proteomics Analyzer (Applied Biosystems, Foster City, CA). Monoisotopic peak masses were acquired in a mass range of 800-4000 Da, with a signal-to-noise ratio (S/N) of 200. Five of the most intense ion signals, excluding common trypsin autolysis peaks and matrix ion signals, were selected as precursors for MS/MS acquisition. The peptide mass fingerprint (PMF) combined MS/MS data were submitted to MASCOT version 3.0 (Matrix Science) for identification according to the NCBInr database (release 16/01/2010, 10343571 sequences, 3528215794 residues). The search parameters were set as follows: Gallus, trypsin cleavage (one missed cleavage allowed), carbamidomethylation of cysteine as fixed modification, oxidation of methionine as variable modification, peptide mass tolerance set at 100 ppm, fragment tolerance set at 0.8 Da. The criterion for successful identification of a protein was a protein score confidence interval (C.I. %) ≥95%.

### Gene Ontology (GO) analysis

Spot identities were submitted to GORetriever (http://www.agbase.msstate.edu/) to obtain the GO annotations. If no annotation was returned, GOanna was used to retrieve GO annotations assigned based on sequence similarity. The resulting annotations were summarized based on the GOA and whole proteome GOSlim set using GOSlimViewer [45].

## Results

### Determination of REV genome load in infected spleens

**Fig. 1:**
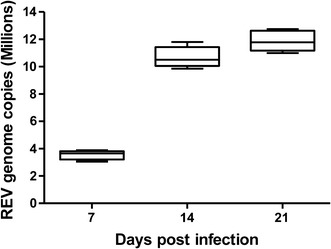
REV genome load in infected spleens. Chickens were infected with the HLJ07I strain of REV and sampled at 7, 14 and 21 days postinfection. Gag copy numbers in 100 ng of spleen RNA were quantitated using real-time RT-PCR. At least three spleen samples were analyzed in duplicate at each sampling time point. The error bars represent standard error of the mean

Successful REV infection was verified using real-time RT-PCR. The results are presented in Figure 1. Virus was not detected in the spleens of chickens in the control group. In the REV-infected group, high levels of viral RNA copies were detected in the spleen at 7, 14, and 21 dpi, indicating the development of persistent infection.

### Spot profiles of 2D PAGE of REV-infected and uninfected control chicken spleens

**Fig. 2:**
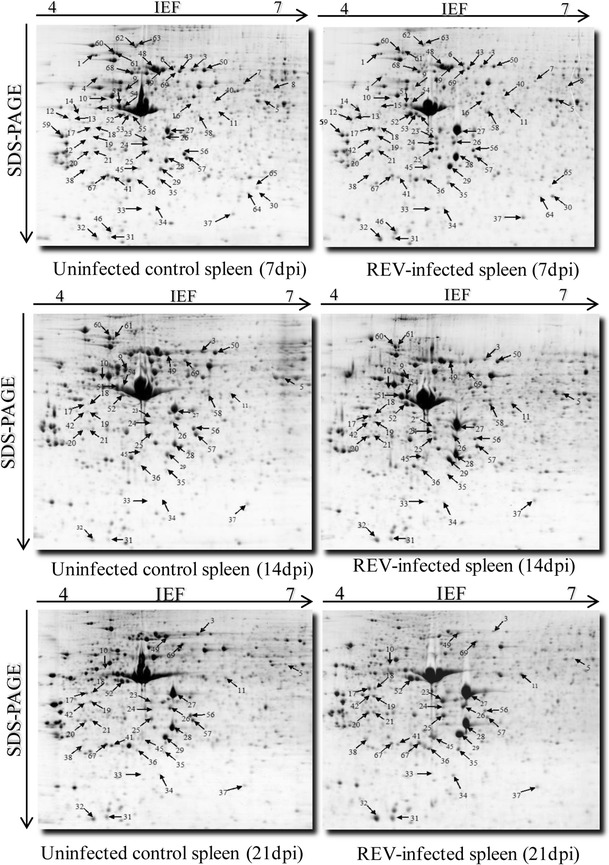
Representative 2D gel images of REV-infected and uninfected control spleen proteomes at different sampling times. The differentially expressed protein spots are shown in gels with their unique sample spot protein numbers. Arrows indicate the successfully identified protein spots. Please refer to Table 1 for the identities of the proteins in the numbered spots

Proteins from spleens of REV-infected and uninfected control chickens at 7, 14 and 21 dpi were extracted and analyzed by 2-DE in order to compare the protein expression profiles of each group. Approximately 500-600 distinct protein spots could be resolved by 2-DE using pH 4-7NL IPG strips loaded with 400 μg of total protein. The molecular weights of the proteins in the spots ranged from 12 to 105 kDa. Both qualitative and quantitative differences were seen in the protein profiles of infected and uninfected chickens (Fig. 2). Comparing multiple 2-DE gel images, we found 70 protein spots in which the proteins were differentially expressed in the infected and uninfected groups at different time points. After statistical analysis, we selected 63 proteins with steady change at three time points for identification by MALDI-TOF/TOF. Sixty of them were successfully identified, including 15 that were persistently upregulated, 14 that were persistently downregulated, and 31 with fluctuating expression (Table 1 and Fig. 2).

**Table 1:** List of the differentially expressed proteins in spleen identified by MALDI/TOF/TOF and MS/MS analysis

Spot ID	Protein name	Accession no.	Fold change in expression			pI, MW (kDa)	Protein score	Function
			7 dpi	14 dpi	21 dpi			
	**Cellular structural protein**							
42	Beta-actin	gi|63018	+2.0	+1.6	+2.3	5.29/42.07	505	Unknown
63	Alpha-actinin-4 (ACTN4)	gi|45384104	-1.4	-1.1	N	5.13/104.71	528	Actin binding calcium ion binding
7	Actin, cytoplasmic type 5	gi|56119084	-2.1	N	N	5.30/42.15	265	ATP binding
17	Actin, cytoplasmic type 5 (ACT5)	gi|56119084	+3.3	+1.5	+2.6	5.30/42.15	235	ATP binding
18	Actin, cytoplasmic type 5 (ACT5)	gi|56119084	+2.4	+1.4	+3	5.30/42.15	694	ATP binding
20	Actin, cytoplasmic type 5 (ACT5)	gi|56119084	+2.8	+1.5	+2.5	5.30/42.15	735	ATP binding
21	Actin, cytoplasmic type 5 (ACT5)	gi|56119084	+1.7	+1.4	+1.9	5.30/42.15	576	ATP binding
22	Actin, cytoplasmic type 5 (ACT5)	gi|56119084	+6.1	+1.9	+3.1	5.30/42.15	868	ATP binding
23	Actin, cytoplasmic type 5 (ACT5)	gi|56119084	+14.9	+5.6	+2.3	5.30/42.15	898	ATP binding
24	Actin, cytoplasmic type 5 (ACT5)	gi|56119084	+5.0	+1.8	+2.1	5.30/42.15	715	ATP binding
25	Actin, cytoplasmic type 5 (ACT5)	gi|56119084	+3.0	+1.2	+2.4	5.30/42.15	888	ATP binding
26	Actin, cytoplasmic type 5 (ACT5)	gi|56119084	+5.2	+1.2	+2.6	5.30/42.15	808	ATP binding
27	Actin, cytoplasmic type 5 (ACT5)	gi|56119084	+2.2	+1.6	+1.8	5.30/42.15	938	ATP binding
28	Actin, cytoplasmic type 5 (ACT5)	gi|56119084	+3.1	+11.5	+2.1	5.30/42.15	781	ATP binding
29	Actin, cytoplasmic type 5 (ACT5)	gi|56119084	+3.6	+1.6	+1.8	5.30/42.15	728	ATP binding
30	Actin, cytoplasmic type 5 (ACT5)	gi|56119084	+9.8	N	N	5.30/42.15	100	ATP binding
31	Actin, cytoplasmic type 5 (ACT5)	gi|56119084	+2.5	+2.1	+2.3	5.30/42.15	587	ATP binding
32	Actin, cytoplasmic type 5 (ACT5)	gi|56119084	+3.7	+2.2	+2.5	5.30/42.15	81	ATP binding
33	Actin, cytoplasmic type 5 (ACT5)	gi|56119084	+2.0	+1.2	+2.1	5.30/42.15	611	ATP binding
34	Actin, cytoplasmic type 5 (ACT5)	gi|56119084	+2.0	N	N	5.30/42.15	650	ATP binding
13	Actin, cytoplasmic type 5	gi|56119084	+3.2	N	N	5.30/42.15	235	ATP binding
36	Actin, cytoplasmic type 5 (ACT5)	gi|56119084	+2.5	+2.5	+1.9	5.30/42.15	741	ATP binding
45	Actin, cytoplasmic type 5 (ACT5)	gi|56119084	+8.9	+9.4	+4.0	5.30/42.15	837	ATP binding
53	Actin, cytoplasmic type 5 (ACT5)	gi|56119084	+1.6	+1.5	+1.3	5.30/42.15	618	ATP binding
58	Actin, cytoplasmic type 5 (ACT5)	gi|56119084	+2.2	+2.5	N	5.30/42.15	523	ATP binding
9	Vimentin	gi|212868	-1.5	-1.8	-6.4	5.09/53.16	177	Structural molecule activity
12	Vimentin	gi|212868	+5.7	N	N	5.09/53.16	795	Structural molecule activity
55	Lamin-B2(LMNB2)	gi|45384202	+3.3	N	N	5.31/68.01	675	Structural molecule activity
	**Signal transduction**							
41	PREDICTED: similar to D4-GDP-dissociation inhibitor (Rho-GDI)	gi|50728568	+1.6	N	N	5.08/22.92	595	Rho GDP-dissociation inhibitor activity
67	Rho GDP-dissociation inhibitor 1 (Rho-GDI)	gi|53126513	-1.6	-1.1	N	5.22/23.31	124	Rho GDP-dissociation inhibitor activity
	**Stress-response and immune-response-associated proteins**							
3	Heat shock protein Hsp70	gi|37590081	-1.9	-1.4	-2.3	5.66/70.09	949	Stress response
6	Heat shock cognate 71 kDa protein (HSPA8)	gi|45384370	+3.0	N	N	5.47/71.01	591	Stress response
16	60 kDa heat shock protein, mitochondrial precursor (Hsp60)	gi|61098372	+2.0	+2.0	N	5.72/61.10	695	Chaperone
19*	Heat shock cognate 71 kDa protein	gi|45384370	+4.3	+2.1	+2	5.47/71.01	38	Stress response
43	Stress-70 protein, mitochondrial precursor	gi|57524986	+1.4	N	+2.2	6.09/73.43	68	Cell proliferation and cellular aging
60	Heat shock protein HSP 90-alpha (Hsp90AA1)	gi|157954047	-3.3	-1.9	N	5.01/84.40	370	ATP binding response to stress
61	Heat shock protein HSP 90-alpha (Hsp90AA1)	gi|157954047	-1.4	-1.8	N	5.01/84.40	544	ATP binding response to stress
10	Cognin/prolyl-4-hydroxylase/protein disulfide isomerase(PDIA3)	gi|21703694	+2.9	+1.2	+1.6	4.75/58.89	477	Catalytic activity
40	Protein disulfide-isomerase A3 precursor (PDIA3)	gi|45383890	+1.8	N	N	5.76/56.54	515	Protein disulfide oxidoreductase activity
	**Calcium ion binding**							
2*	Parvalbumin (pvalb1)	gi|225877920	+2.7	N	N	4.94/12.06	44	Calcium ion binding
50	Annexin A6 (ANXA6)	gi|50982399	-2.1	-1.4	-2.1	5.57/75.57	180	Calcium ion binding
56	Chain A, Crystal Structures Of Chicken Annexin V	gi|62738641	-1.6	-1.4	-1.7	5.61/36.15	680	Calcium ion binding
	**Apoptosis or tumor-associated proteins**							
15	Serpin B6 (SERPINB6)	gi|57530448	+2.3	N	N	5.28/43.24	397	Serine-type endopeptidase inhibitor activity
70	Cathepsin B (CTSB)	gi|603203	+2.0	+1.4	+1.3	5.74/38.47	144	Regulation of catalytic activity
62	Valosin containing protein	gi|90990971	-4.0	N	-1.48	5.14/89.95	636	Nucleoside-triphosphatase activity
37	Ferritin H subunit	gi|21177	+2.3	+6.6	+1.3	5.78/21.24	493	Ferroxidase activity
	**Other**							
1	Gamma-glutamyltransferase (TGL2)	gi|62903517	-2.7	N	N	4.90/79.11	875	Gamma-glutamyltransferase activity
5	Argininosuccinate synthase(ASS1)	gi|61657937	+2.1	+1.4	N	6.10/47.33	872	Arginine succinate synthetase activity
8	Pre-mRNA-processing factor 19 (PRPF19)	gi|86129600	-1.9	N	N	6.19/55.55	394	DNA binding DNA repair
14	PREDICTED: ribosomal protein SA isoform 1(RPSA)	gi|118086026	+3.8	N	N	4.8/33.11	211	Laminin receptor Activity(*Rattus norvegicus*)
48*	Otokeratin	gi|3746660	+1.7	N	N	5.97/53.77	56	Structural molecule activity
49	Serum albumin precursor (VTDB)	gi|45383974	-1.3	N	-2.8	5.51/71.86	341	Vitamin transporter activity
57	Chain B, Solution Nmr Structure Of V-1 Bound To Capping Protein	gi|297787504	-1.7	N	-1.4	5.5/30.71	457	Actin binding
59	Alpha-tropomyosin (smooth muscle)	gi|212807	+1.6	N	+4.9	4.68/32.89	292	Actin binding
65	Similar to MGC84496 protein	gi|118101652	-1.5	-1.4	-2.3	6.07/22.80	176	Unknown
	**Hypothetical proteins**							
4	PREDICTED: hypothetical protein, partial	gi|118105147	+1.1	+1.7	+1.4	4.77/66.11	147	Unknown
11	Hypothetical protein	gi|53136508	+2.0	+1.2	+1.9	6.22/53.74	257	Unknown
38	Hypothetical protein	gi|53126859	+13.8	+7.2	+6.0	5.31/42.10	535	Unknown
35	Hypothetical protein	gi|53126859	+4.1	+3	+2.9	5.31/42.10	650	ATP binding
51	Hypothetical protein	gi|53126859	-1.2	-1.4	-1.2	5.31/42.10	852	Uknown
52	Hypothetical protein	gi|53126859	+2.6	+1.3	+1.7	5.31/42.10	881	Unknown
54	Hypothetical protein	gi|53126859	-2.0	-2.5	-1.6	5.31/42.10	676	Unknown
69	Hypothetical protein	gi|60098585	-1.8	-1.2	-1.6	5.53/60.19	850	Chaperone ATP binding

**Fig. 3:**
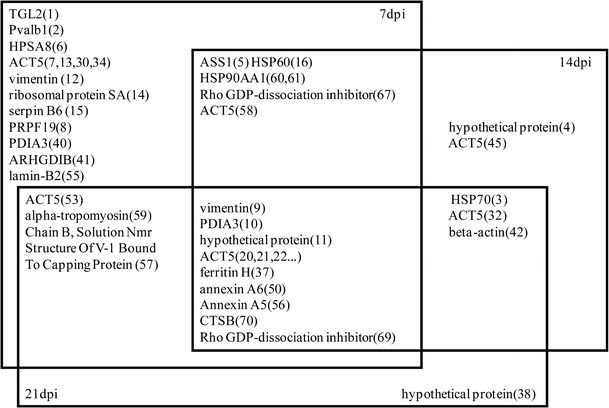
Venn diagram summarizing the spots that were significantly differentially expressed in the spleen tissues of REV-infected chickens according to their corresponding time of sampling. The identities of spots that were commonly expressed were placed in overlapping areas accordingly. The corresponding spot numbers are shown in parentheses. Refer to Table 1 for the respective protein names

a) Spot ID is the unique number that refers to the labels in Figure 3

b) Accession number: gi number in NCBI

c) Score: protein score based only on MS spectra by MALDI-TOF. Other spots were based on combined MS and MS/MS spectra from MALDI-TOF-TOF identification. A protein score greater than 83 is significant in this study (*P* < 0.05)

“-”, downregulated protein; “+”upregulated protein; “N”, no difference; “*”, failed identification

### Identification of differentially expressed proteins by mass spectrometry

In order to identify differentially expressed proteins, the spots were manually excised from preparative gels prepared by loading 400 μg of total protein and staining with Coomassie blue. Subsequently, trypsin-digested spots were identified by MALDI-TOF-TOF MS. The PMF data was searched against the NCBInr database using MASCOT. In total, the identity of proteins in 60 different spots representing 28 different proteins was determined (Table 1). Moreover, several spots contained peptides generated from multiple proteins.

Several proteins were differentially expressed at more than one time point, e.g., vimentin, cognin/proly1-4-hydroxylase/protein disulfide isomerase (PDIA3), hypothetical protein, actin-cytoplasmic type 5 (ACT5), ferritin H subunit, annexin A6 (ANXA6), annexin A5 (ANXA5), cathepsin B (CTSB), and rho GDP-dissociation inhibitor 1 (Rho-GDI) at all three time points. In total, 14 proteins were differentially expressed exclusively at 7 dpi. There was only one spot that was specific to 21 dpi (Fig. 3). The significant changes detected with the rest of the spots overlapped between time points to varying degrees. Differences in the pattern of change were observed predominantly in the early stages of the infection cycle, which suggests that critical events early in infection are important in determining the fate of the host.

Interestingly, some of the spots representing a particular protein showed opposite directions of regulation even within the same group. For example, three spots with different molecular weights and pI values were identified as hypothetical proteins (Table 1). Of these three spots, two spots with molecular weights of 53.74 and 42.10 kDa were found to be upregulated in infected chickens at more than one time point. The summarized distribution of the identities of spots identified at each time point is presented in Figure 3.

### Gene Ontology annotation

**Fig. 4:**
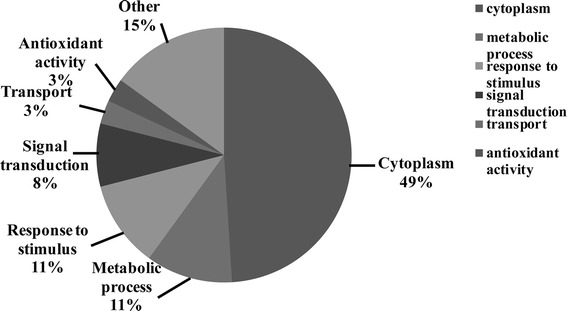
Gene Ontology analysis of significantly changed proteins according to their biological process. This classification was produced based on the analysis using the GOSlimViewer tool at the Agbase database (http://www.agbase.msstate.edu/) as described in Materials and methods

In order to gain an overview of the subcellular location and biological processes associated with proteins that underwent changes in expression levels in REV-A infected spleen, categorization of these proteins was performed based on Gene Ontology (GO) annotations. As shown in Figure 4, 49% of these proteins were identified as cytoplasmic (term GO: 0005737). The most frequent associated biological process GO terms were metabolic process (GO: 0008152) and response to stimulus (GO: 0050896). These was followed by GO terms including but not limited to signal transduction, transport and antioxidant activity.

## Discussion

In the present study, we have profiled the global protein expression changes in chicken spleen that occur in response to REV infection at various time points representing different phases of the REV life cycle. Furthermore, we have identified proteins that were differentially expressed between infected and uninfected birds, using mass spectrometry.

Among the 28 proteins that were differentially expressed, there was a considerable number of proteins that produced multiple spots in 2D gels. In addition, there was some degree of disagreement between the expected and experimental molecular weights for some of the proteins. There could be several reasons for this. First, some proteins exist as different isoforms (and therefore have different pI values) as well as different intermediate stages between translation and attainment of the functionally mature form (i.e., pre-pro- and pro-forms of the protein). This could change the molecular weight and/or the isoelectric point as discussed below. Another reason is possible protein degradation between sample collection and processing.

Analysis of the virus load revealed high levels of viral RNA copies in spleen tissue at 7, 14, and 21 dpi, indicating the development of persistent infection. The virus load reflects the status of viral replication and disease evolution, and it has therefore been used as an important marker for disease progression of retroviral infections [57, 74]. Thus, it is conceivable that the virus copy numbers observed in the present study would be indicative of a high level of infection.

The proteins identified in the present study are involved in a variety of cellular processes, notably, formation of the cytoskeleton, cellular metabolism, response to stimulus, signal transduction, transport and antioxidant activity. The significance of these processes in chicken-REV interactions is discussed below. Although not determined in the present study, temporal changes in the cellular composition of the spleen may have, at least in part, influenced the organ proteome of the whole spleen. However, the advantage of our method is that the proteins that are differentially expressed could be used to point to cell subsets and/or mechanisms that have a potential involvement in REV-host interactions and could be targeted for future studies.

### Alterations of cytoskeletal proteins after REV-A infection

Cytoskeletal proteins play important roles in maintaining cell morphology, regulating the progress of gene transcription and protein synthesis, and are closely related to intracellular transport and cell division [46]. It has been shown recently that various viruses manipulate and utilize the host cytoskeleton to promote viral infection [56]. Changes in cytoskeleton proteins have been described previously in infections with several other virus, such as infectious bursal disease virus (IBDV) [81], severe acute respiratory syndrome (SARS)-associated coronavirus [36], and human papillomavirus type 8 [2]. In the current study, altered spot profiles were observed for a number of cytoskeleton-associated proteins representing two main categories of cytoskeleton proteins, namely microfilaments and intermediate filaments. Among the microfilament proteins, all detected actin spots, except for alpha-actinin-4 (ACTN4), were significantly upregulated across the experimental period. Intermediate filament, lamin B2 (LMNB2) spots were also upregulated in infected birds at 7 dpi.

Actin, one of the most highly conserved proteins in eukaryotic cells, forms microfilaments, which are one of the three main components of the cytoskeleton. The role of the actin cytoskeleton in virus-induced signaling has been increasingly recognized because it is believed to act as a regulator in gene transcription [46] and might facilitate the efficient spread of progeny virus particles [16, 73]. Actin might also participate in endocytosis, with actin rearrangements contributing to the internalization of virus particles [54]. It has been reported that expression of actin, cytoplasmic type 5(ACT5), a third cytoplasmic isoform of the chicken non-muscle actin [6], is induced in the late stages of IBDV infection [81]. In this study, chickens infected with REV showed high levels of ACT5 at 7, 14 and 21 dpi, demonstrating that ACT5 may play important roles in REV infection and proliferation.

Another component of the cytoskeleton that displays considerable alteration in chickens infected with REV is lamin B2, which forms intermediate filaments. In birds, three structurally distinct lamin isotypes have been identified according to the homologies of their amino acid sequences [69] and their protein chemical properties [40, 62]; they have been designated as lamins A, B1, and B2. Lamin B2 is the quantitatively predominant B-type lamin in chickens [30]. Previous studies have suggested that lamin is essential for DNA replication [13]. Therefore, upregulation of lamin-B2 at 7 dpi would be beneficial for REV replication.

Viruses require cytoskeletal proteins for viral entry and establishment of infection [49], and the disruption of vimentin might block virion assembly and budding [34, 72]. Several studies have shown that human immunodeficiency virus (HIV) type 1 protease cleaves the intermediate filament protein vimentin and induces the collapse of vimentin in infected cells [34, 60]. We found that vimentin was downregulated. Further studies are required to determine whether REV uses an HIV-like strategy to cleave vimentin, resulting in highly decreased expression and the collapse of the vimentin network.

Herpesviruses are known to interact with the actin filament system and its regulatory protein, Rho GTPase, at various stages of infection [21]. Cumulative evidence also shows that the GTPases of the Rho family are key regulatory molecules of the actin cytoskeleton [29]. Rho-GDP-dissociation inhibitor 1 (Rho-GDI) has also been reported to be member of the family of Rho-GTPase regulators, which regulate a wide variety of cellular functions by binding and inhibiting Rho GTPases [68]. Thus, it is hypothesized that downregulation of Rho-GDI during REV infection may interfere with GTPase activity.

### Apoptosis or tumor-associated proteins

Cathepsin B (CathB), a cysteine proteases commonly found in lysosomes, was among the proteins that were differentially expressed in infected spleens at all three time points. The functions of cathepsin B have been linked to cell proliferation, cell differentiation, organogenesis, metabolic processes, and progression of various human tumors [79]. Various studies have suggested that cathepsin B participates in the pathology of carcinomas, and it is present at increased levels in advanced tumor stages [19, 61]. Therefore, it is conceivable that upregulated CathB may play a role in tumor formation after REV infection; however, this needs to be studied further.

The annexins are a family of Ca^2+^- and phospholipid-binding proteins that interact with membranes when there is an increase in the Ca^2+^ concentration or during cytoplasmic acidification [8]. Annexin A5 represents a typical member of this protein family and is an important modulator of the immune response against apoptotic cells, necrotic cells, and certain viruses [48]. Annexin A6 (AnxA6) is also a member of this protein family that have been implicated in an array of physiological processes such as cell proliferation, differentiation, and signal transduction [25, 27]. In this study, the abundance of annexin A5 and annexin A6 was shown to be decreased at 7, 14 and 21 dpi, which suggests that those proteins may play special roles during REV infection or replication.

Members of the serpin superfamily are protease inhibitors and are associated with cell death/apoptosis and protect cells from protease-mediated cell injury and death by inhibiting the activation of serine proteases [5, 44]. Serpin B6, which is largely involved in immunity, is an intracellular serpin expressed primarily in myeloid cells, platelets, endothelial and epithelial cells [59]. It has been reported that serpin B6 plays an important role in HIV replication [82]. The high expression levels of serpin B6 in REV-infected chickens may be relevant to the reticuloendotheliosis caused by REV.

Ferritin is a 24-subunit protein composed of two subunit types, termed H and L. The ferritin H subunit has a potent ferroxidase activity that catalyses the oxidation of ferrous iron, whereas ferritin L plays a role in iron nucleation and protein stability [52]. Ferritin plays a role in protecting endothelial cells [3] and tumor cells [10] from oxidant damage. Increasing ferritin levels always accompany or closely precede rapid disease progression in HIV-infected patients [20]. In this study, chickens infected with REV showed high levels of ferritin at 7, 14 and 21 dpi, which may indicate advanced or progressive disease.

### Stress-response- and immune-response-associated proteins

Heat-shock proteins (HSPs), which are expressed constitutively in all cells, are essential for several important cellular processes, such as protein folding, protection of proteins from denaturation or aggregation, facilitation of protein transport, and stimulation of innate and adaptive immune responses [71]. Several stress proteins have been shown to play roles in the life cycle of a variety of RNA and DNA viruses [58]. In this study, four HSPs were differentially expressed during REV infection. One was HSP60, a highly conserved stress protein that has chaperone functions in regulating apoptosis [26] and eliciting a potent proinflammatory response in cells of the innate immune system [51]. Two others were HSP70 and HSC70, which are involved in apoptosis regulation, signal transduction and eliciting cancer immunity [4, 18, 67]. Hsp70 induction serves to signal to the immune system the presence of an immunologically relevant (dangerous) situation against which an immune reaction should be raised [65]. Both the stress-inducible protein Hsp70 and its constitutive form Hsc70 interact with various viral proteins and may be involved in the assembly of adenovirus, enterovirus and polyomavirus capsid protein complexes [15, 42, 43]. Hsp70 expression is selectively increased following infection with HIV-1 [70], and it has been suggested that Hsp70 plays a role in the nuclear import of HIV-1 preintegration complexes [1] and early events of infection [28]. The fourth HSP was HSP90, the expression of which was reduced in REV-infected chickens at 7 and 14 dpi. Hsp60, Hsp70, and Hsp90 have been shown to interact with hepatitis B virus reverse transcriptase and to facilitate the initiation of viral DNA synthesis from hepatitis B virus pregenomic RNA [35, 53]. Significant changes in the expression of HSP70 and HSP90 in the spleen of MDV-infected birds has been reported [64]. In line with that, we also observed downregulated expression of HSP90 proteins in REV-infected chickens. However, the possible effects of HSP90 on immune suppression caused by REV needs to be explored in future studies.

Protein disulfide isomerase A3 (PDIA3) is a member of the endoplasmic reticulum stress signaling pathway and is associated with malignant stages of prostate cancer [50, 55]. In addition to its role in the ER stress pathway, PDIA3 is a part of the major histocompatibility complex (MHC) class I peptide-loading complex, which is essential for the formation of the final antigen conformation and for export from the endoplasmic reticulum to the cell surface [24]. In PDIA3-deficient mice, this complex is impaired and negatively influences presentation of antigenic peptides. This may help tumors to escape from immune surveillance by cytotoxic T cells [23]. PDIA3 aides in dengue virus (DENV) replication by suppressing TNF-α production in human monocytic THP1 cells [47]. Our previous studies showed that TNF-α was also downregulated in REV-infected chickens [78]. Thus, the upregulation of PDIA3 may relevant to tumor formation and contribute to REV-A replication.

### Hypothetical proteins

Hypothetical proteins are predicted proteins that do not have experimental evidence for their translation nor do they have a characterized function [22]. In this study, hypothetical protein RCJMB04_1h13 with ID gi|53126859 was highly increased at 7, 14 and 21 dpi. As reported by Caldwell et al., the most frequent molecular function associated with a domain is ‘ATP binding’, assigned to protein kinase domains and other domains such as the AAA ATPase family, ABC transporters and others [9]. The upregulation of hypothetical proteins is similar to that of actin in this study, which demonstrates that they may play important roles in REV infection and proliferation.

In conclusion, the findings of the present study highlight some of the mechanisms involved in the host response in the spleen to REV infection at various time points representing different stages of REV pathogenesis. Although the functions of the proteins that were identified here were not studied, it is likely that all or some of them are involved in host-virus interactions. One of the limitations of the tools used in this study is the inefficiency of detecting low-abundance proteins or those with low molecular weights, such as cytokines and chemokines. Therefore, a more comprehensive study is needed to elaborate on our present observations and to further explore other proteins that may play a role in the pathogenesis of and host responses to this virus.
